# Supplementary material for: Essentiality of sterol synthesis genes in the planctomycete bacterium Gemmata obscuriglobus
Source: Nat Commun. 2019 Jul 2;10:2916. doi: 10.1038/s41467-019-10983-7 (PMC6606645; doi:10.1038/s41467-019-10983-7)
Supplement: Supplementary file 1 — Supplementary Information [file 41467_2019_10983_MOESM1_ESM.pdf]

Supplementary Information for

Essentiality of sterol synthesis genes in the planctomycete bacterium *Gemmata obscuriglobus*

Rivas-Marín, E. *et al.*

This PDF file includes:

Supplementary Figs. 1 to 7
Supplementary Tables 1to 3
Supplementary References

Other supplementary materials for this manuscript include the following:

Supplementary Movies 1 to 2

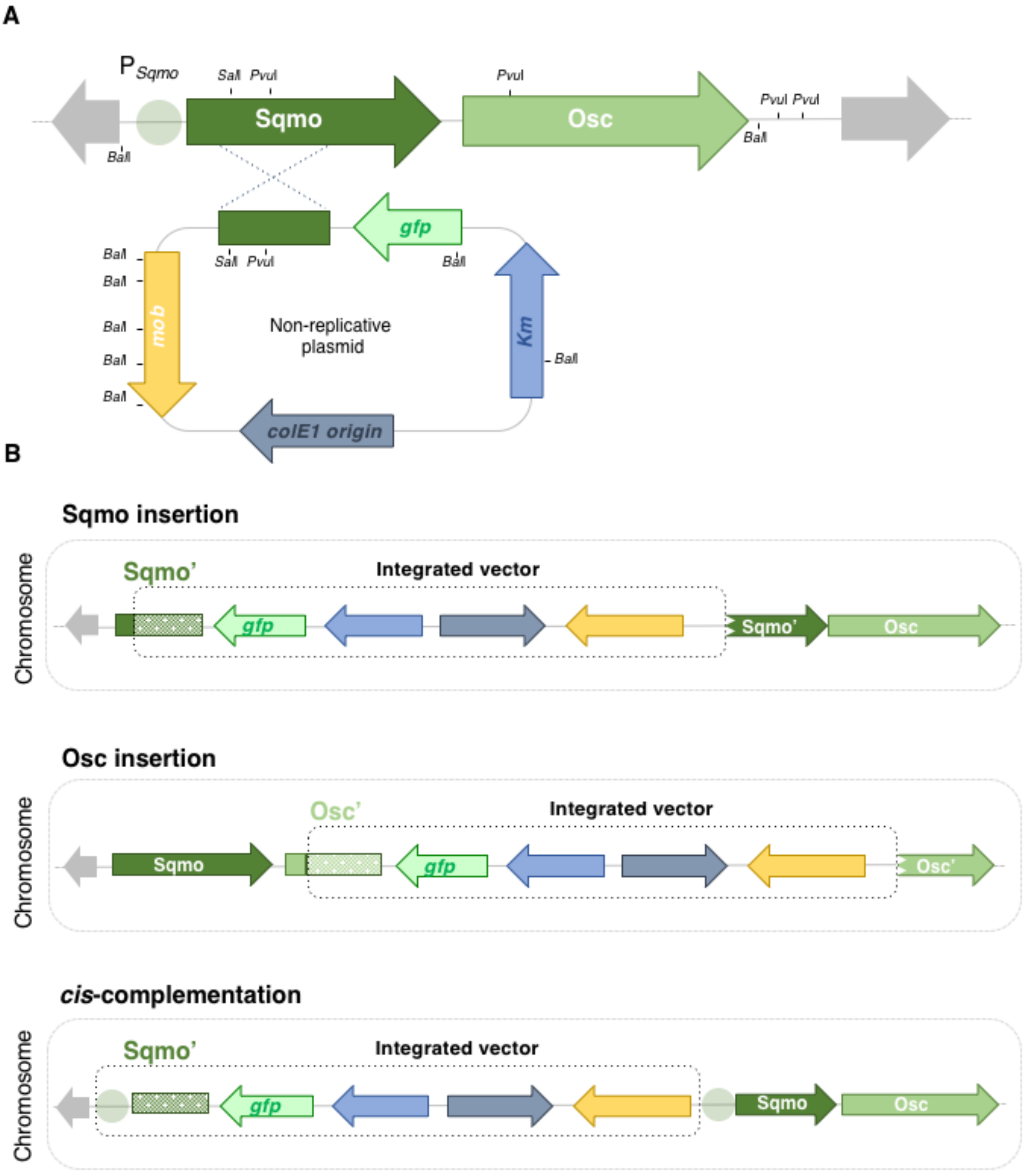

Supplementary Figure 1. Schematic representation of *Gemmata obscuriglobus* mutant construction strategy. (*A*) Genomic organization of genes required to synthesize sterols in *G. obscuriglobus* and plasmids used for mutagenesis. The fragment cloned into the plasmid for the homologous recombination varied depending on the mutant constructed. Restriction sites for the Southern blotting assay (Supplementary Fig. 2) are indicated. (*B*) Resulting genomic organization of the mutant strains after plasmid insertion. *sqmo* insertion mutant (DV006) was the result of pDV011 plasmid integration; *osc* insertion mutant (DV042) resulted from pDV058 plasmid integration; *cis*-complemented *sqmo* mutant (DV026) was the result of pDV037 plasmid integration.

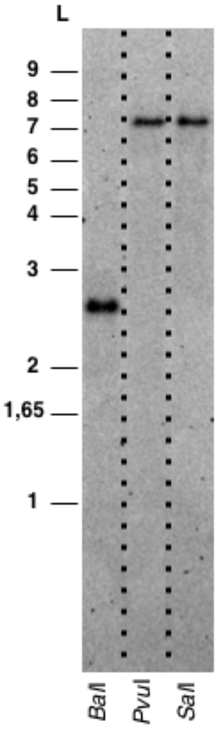

Supplementary Figure 2. Southern-blotting analysis of the *Gemmata obscuriglobus* DV026 strain. The *gfp* gene harbored in the plasmid integrated into the genome was used as a probe. The expected sizes for the *Bal*I, *Pvu*I and *Sal*I restriction fragments were 2257 7474, and 7474 bp, respectively.

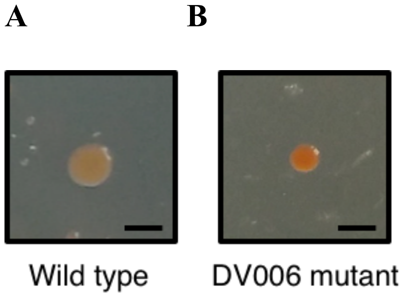

Supplementary Figure 3. Colony phenotype of wild type and *sqmo* mutant. (*A*) Wild-type colony, (*B*) DV006 mutant colony. Scale bars are 0.1 cm.

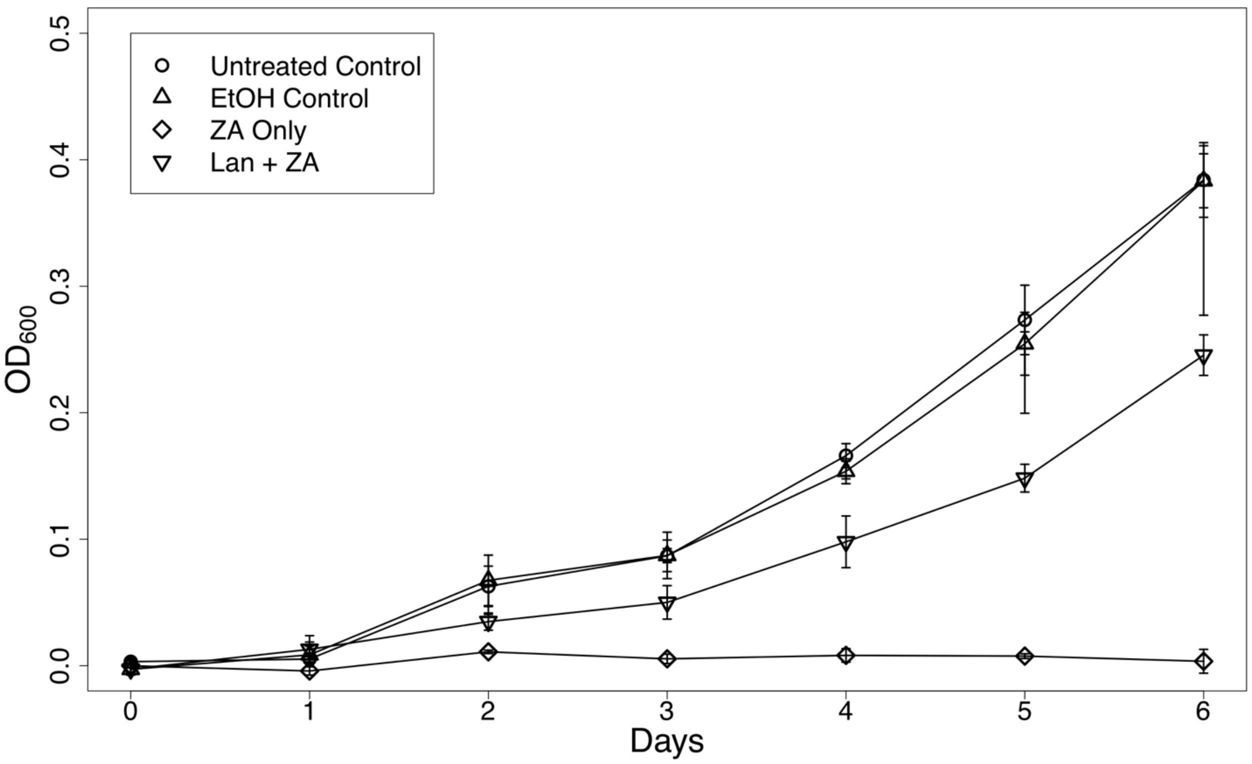

Supplementary Figure 4. Inhibition and rescue of *Gemmata obscuriglobus* growth through treatment with zaragozic acid and lanosterol. Growth was measured spectrophotometrically over a 6-day time-course. Means and standard deviations are shown for five biological replicates. Points are represented by shapes, where circles represent the untreated control, triangles represent the ethanol control, diamonds represent the zaragozic acid (ZA) treatment, and upside-down triangles represent the zaragozic acid and lanosterol (Lan) treatment.

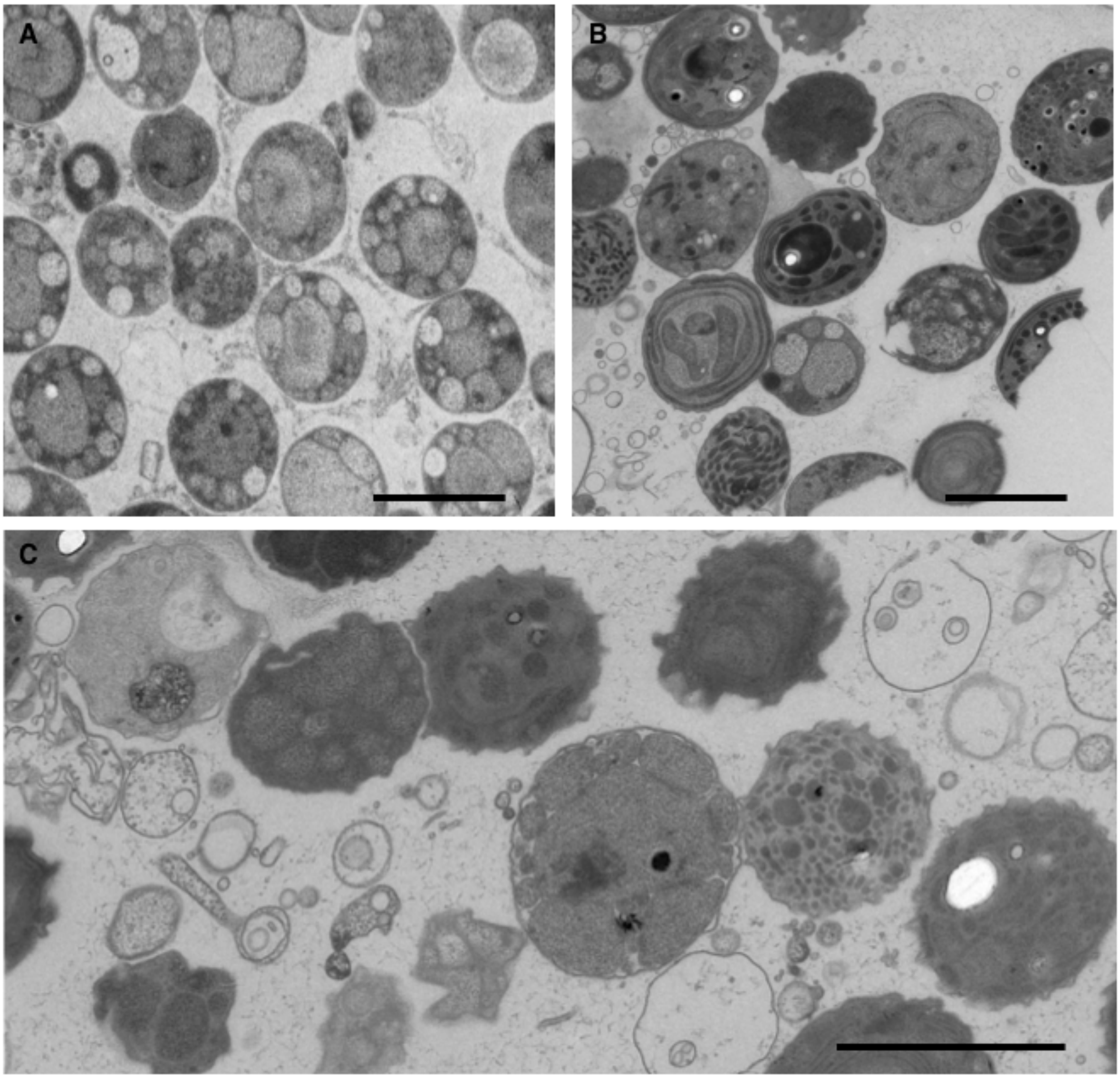

Supplementary Figure 5. Wide-field overview of electron micrographs of wild-type and *sqmo* mutant cultures. (*A*) Wild-type culture overview, (*B*) representative membrane organizations in the *sqmo* mutant, (*C*) cell debris are abundant in *sqmo* mutant culture. Scale bars are 2 μm.

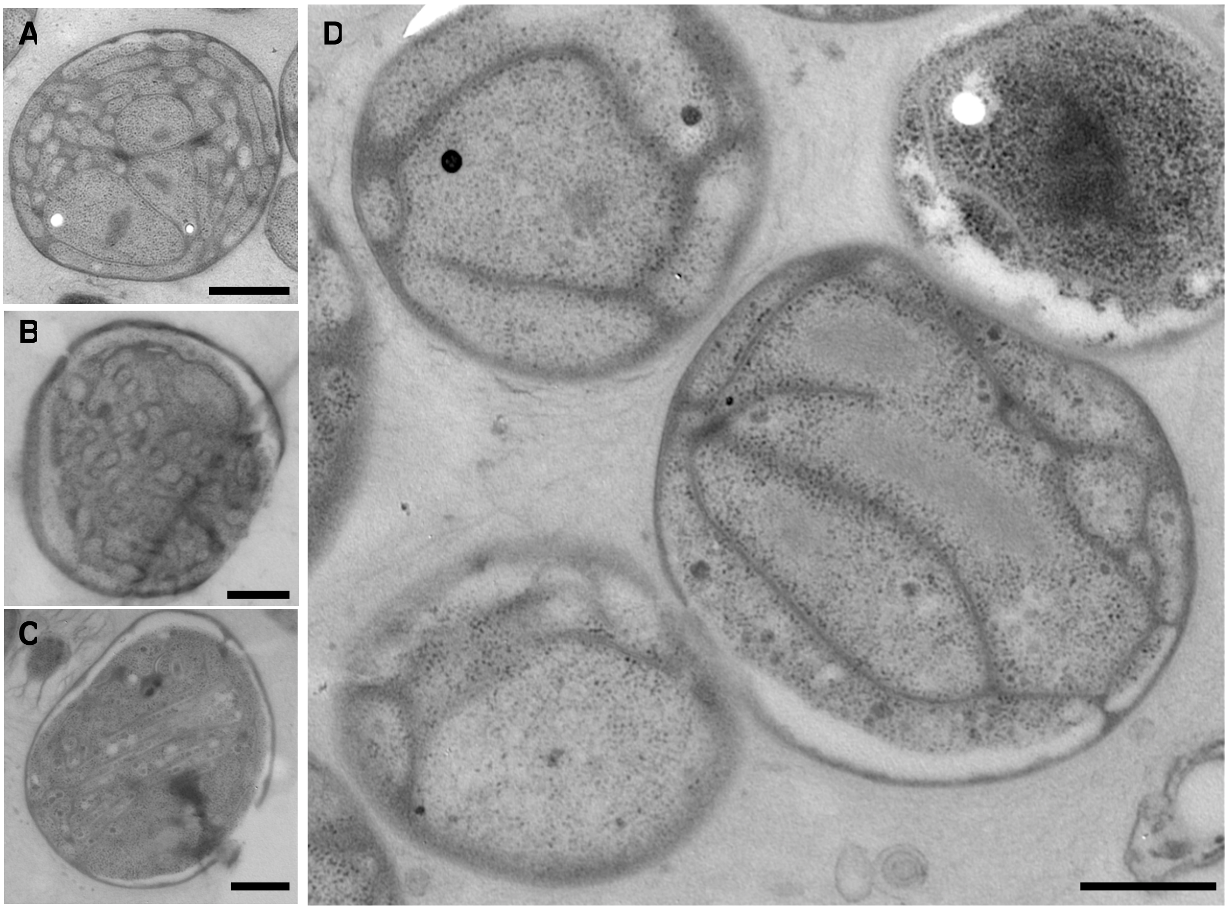

Supplementary Figure 6. Transmission electron microscopy of *Gemmata obscuriglobus* wild-type cells treated with terbinafine. Representative membrane phenotypes observed in the *G. obscuriglobus* mutant upon terbinafine treatment. Various membrane organizations are shown (*A-D*). Scale bars are 0.5 μm.

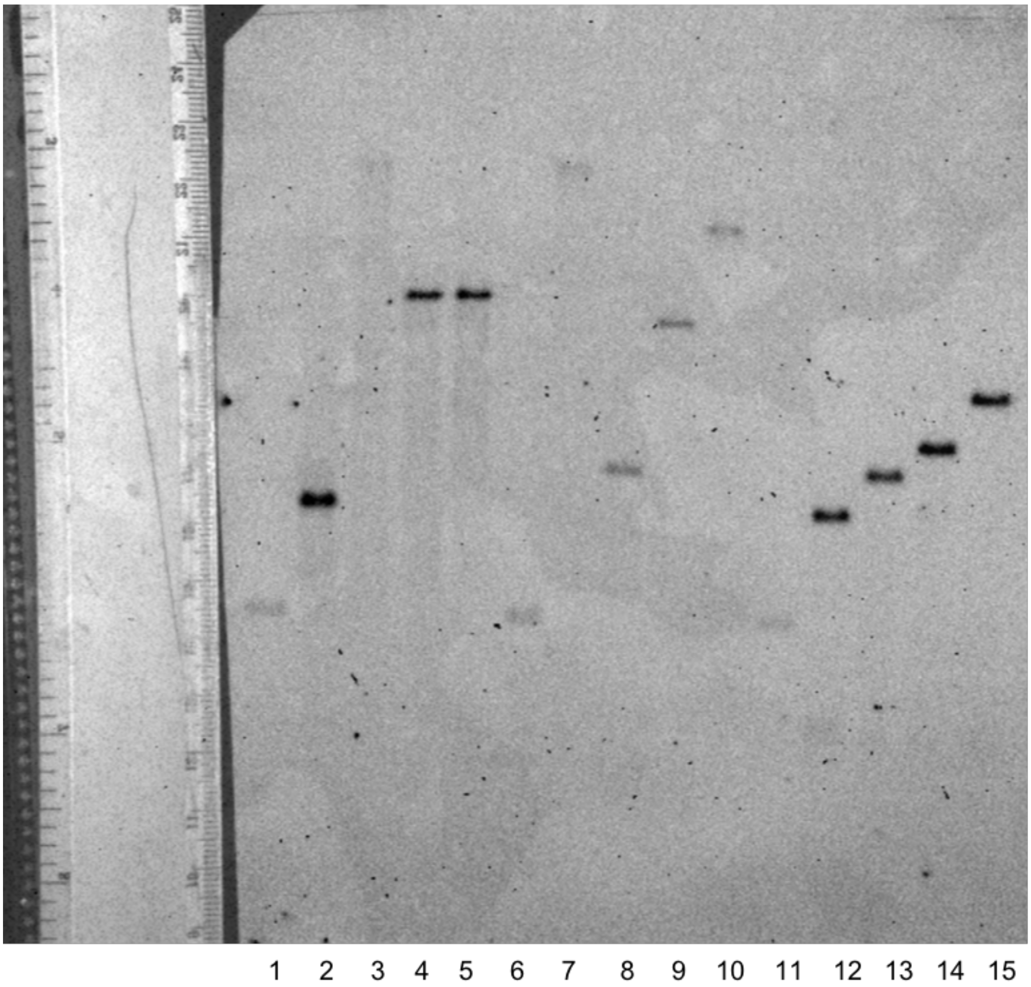

Supplementary Figure 7: Extended southern-blotting analysis of the *Gemmata obscuriglobus* DV026 strain. Source data Supplementary Figure 2. Line 1 and 6 show the ladder where 1,65 kbp band is visible. Lines 2 to 5 show the *Bal*I, *Hind*III, *Pvu*I and *Sal*I restrictions. *Hind*III digestions (not shown in Figure S2) has expected size bigger than 17 kbp. Lines 7 to 15 are unrelated to this manuscript.

Supplementary Table 1. Strains used in this study.

Strain name	Genotype	Reference
Escherichia coli DH5α	F^- ϕ80*lacZ*ΔM15 Δ(*lacZYA-argF)U169* *recA1* *endA1* *hsdR17*($r_K^- m_K^-$) *supE44* *thi-1* *gyrA* *relA1*	(1)
Gemmata obscuriglobus DSM5831^T	Wild-type strain	(2)
Gemmata obscuriglobus DSM5831^T DV006	*sqmo*'::pDV011	This work
Gemmata obscuriglobus DSM5831^T DV042	*osc*'::pDV058	This work
Gemmata obscuriglobus DSM5831^T DV026	P_{sqmo}-*sqmo*'::pDV037	This work

Supplementary Table 2. Oligonucleotides used in this study

**Primer name**	**Sequence**
Int SQMO *Gemmata* fwd	GGTCAAGCTTTCGCGTTAGCGCGGATGTC (*Hin*dIII)
Int SQMO *Gemmata* rv	CTTGAAGCTTCAAATCCGGCAACGGCCC (*Hin*dIII)
Int SQMO *Gemmata* 2 fwd	GCTCTACGGTTCCGAGTG
Int SQMO *Gemmata* 2 rv	GGGATCGTGCGGCATCAG
Int OSC *Gemmata* fwd	GGTCAAGCTTTCGGCTTGCACCCGGAACACC (*Hin*dIII)
Int OSC *Gemmata* rv	CTTGAAGCTTCGCGTCAGCATGAACTCGGCG (*Hin*dIII)
GFP pDV020 fwd	GGTCGATATCAGTACTGAATTCATGAGTAAAGGAGAAGAACTTTTC
GFP pDV020 rv	CTTGAAGCTTGGGCCCTTTGTATAGTTCATCCATGCCATG

Underlined sequences show restriction sites.

Supplementary Table 3. Plasmids used in this study

Plasmid name	Main features	Source
pMPO1012	Mobilizable, ColE1, Km^r, containing the mut3a-gfp expressed under a heterologous promoter.	(3)
pRK2013	Helper plasmid. ColE1. Tra^+, Km^r	(4)
pDV011	1232 bp fragment in pMPO1012, bearing a *sqmo′* gene from *G. obscuriglobus*. Km^r.	This work
pDV037	1511 bp fragment in pMPO1012, bearing a *sqmo′* gene under its own promoter from *G. obscuriglobus*. Km^r.	This work
pDV058	1054 bp fragment in pMPO1012, bearing a *osc′* gene from *G. obscuriglobus*. Km^r.	This work

Supplementary References

1. Hanahan D (1983) Studies on transformation of Escherichia coli with plasmids. *J Mol Biol* 166(4):557–580.

2. Franzmann P, Skerman V (1984) Gemmata obscuriglobus, a new genus and species of the budding bacteria. *Antonie Van Leeuwenhoek* 50(3):261–268.

3. Rivas-Marín E, Canosa I, Santero E, Devos DP (2016) Development of Genetic Tools for the Manipulation of the Planctomycetes. *Front Microbiol* 7:914.

4. Figurski DH, Helinski DR (1979) Replication of an origin-containing derivative of plasmid RK2 dependent on a plasmid function provided in trans. *Proc Natl Acad Sci U S A* 76(4):1648–1652.
