# Supplementary material for: Essentiality of sterol synthesis genes in the planctomycete bacterium Gemmata obscuriglobus
Source: Nat Commun. 2019 Jul 2;10:2916. doi: 10.1038/s41467-019-10983-7 (PMC6606645; doi:10.1038/s41467-019-10983-7)
Supplement: Supplementary file 4 — Description of Additional Supplementary Files [file 41467_2019_10983_MOESM4_ESM.pdf]

Description of Additional Supplementary Files

Supplementary Movie 1: Time-lapse phase-contrast microscopy of terbinafine-treated *G. obscuriglobus* cells. The video captures a representative field of cells treated with 100 mg/mL terbinafine, showing development of phase-bright inclusions over the 14-hour course of the time-lapse experiment. Inclusion development can be observed in some cells within 1 hour of the beginning of the experiment, and in many cells by the end of the movie. Arrested cell division is also evident in cells containing these inclusions, although cells lacking inclusions continue to divide normally. Video frames were taken at 30-minute intervals. Scale bar is equal to 5 mm.

Supplementary Movie 2: Time-lapse phase-contrast microscopy of terbinafine-treated *G. obscuriglobus* cells supplemented with exogenous lanosterol. The video captures a representative field of cells treated with 100 mg/mL terbinafine and 1 mg/mL lanosterol, showing an apparent rescue effect of terbinafine-induced inclusion development and arrested cell division (Supplementary Movie 1) by lanosterol supplementation. Phase-bright inclusions are not observed, and cell division proceeds normally, as seen in cells not subjected to terbinafine treatment (Fig. 3A). Cells were treated with both terbinafine and lanosterol prior to growth under the microscope, as lanosterol could not be incorporated homogeneously into the agarose pad used for the time-lapse experiment. Video frames were taken at 30-minute intervals. Scale bar is equal to 5 mm.
